# Evaluating spatial patterns of seasonal ozone exposure and incidence of respiratory emergency room visits in Dallas-Fort Worth

**DOI:** 10.7717/peerj.11066

**Published:** 2021-04-13

**Authors:** Kari Northeim, Constant Marks, Chetan Tiwari

**Affiliations:** 1 Department of Biological Sciences, University of North Texas, Denton, TX, USA; 2 Department of Computer Science and Engineering, University of North Texas, Denton, TX, USA; 3 Department of Geography and the Environment, University of North Texas, Denton, TX, USA; 4 Advanced Environmental Research Institute, University of North Texas, Denton, TX, USA

**Keywords:** Air pollution, Respiratory health, Dallas-Fort Worth, Urban pollution, Ozone, Spatial interpolation, Environmental impacts, Emergency room visits

## Abstract

**Background:**

In urban environments, environmental air pollution poses significant risks to respiratory health. Moreover, the seasonal spatial variability of the air pollutant ozone, and respiratory illness within Dallas-Fort Worth (DFW) is not well understood. We examine the relationships between spatial patterns of long-term ozone exposure and respiratory illness to better understand impacts on health outcomes. We propose that this study will establish an enhanced understanding of the spatio-temporal characteristics of ozone concentrations and respiratory emergency room visits (ERV) incidence.

**Methods:**

Air pollution data (ozone) and ERV incidence data from DFW was used to evaluate the relationships between exposures and outcomes using three steps: (1) develop a geostatistical model to produce quarterly maps of ozone exposure for the DFW area; (2) use spatial analysis techniques to identify clusters of zip codes with high or low values of ozone exposure and respiratory ERV incidence; and (3) use concentration-response curves to evaluate the relationships between respiratory ERV incidence and ozone exposure.

**Results:**

Respiratory ERV incidence was highest in quarters 1 and 4, while ozone exposure was highest in quarters 2 and 3. Extensive statistically significant spatial clusters of ozone regions were identified. Although the maps revealed that there was no regional association between the spatial patterns of high respiratory ERV incidence and ozone exposure, the concentration-response analysis suggests that lower levels of ozone exposure may still contribute to adverse respiratory outcomes.

## Introduction

Despite significant efforts to reduce ozone air pollution, urban counties across the United States (US) are in non-attainment of federal standards for the health hazardous pollutant ozone (U.S. Environmental Protection Agency (EPA), 2017). Tropospheric or surface ozone is one of the six criteria pollutants established by the EPA which are monitored and measured according to specific standards (U.S. Environmental Protection Agency (EPA), 2021a). Its formation is part of a secondary mixing chemical reaction created by nitrogen oxides (NOx) and volatile organic compounds (VOC) in the presence of solar radiation (U.S. Environmental Protection Agency (EPA), 2021a). The National Ambient Air Quality Standards (NAAQS) outline the allowable exposure levels. In 2015 the EPA revised the standard levels to 0.070 parts per million (ppm) for the 8-h standard from the previous standard of 0.075 ppm in 2012 (U.S. Environmental Protection Agency (EPA), 2021b). Individual counties are then in compliance or non-compliance with the EPA standard based on a 4th highest daily maximum 8-h average over 3 years (U.S. Environmental Protection Agency (EPA), 2021b). Primary emissions sources found in urban environments that are precursors to ozone formation include: road transportation, power generation and industrial manufacturing (Frumkin, 2002; Field et al., 2015). Further, urban environments demonstrate spatial variation of ozone, but the largest production of ground level ozone tends to be prevalent downwind of known sources and urban areas (Frumkin, 2002; Bell et al., 2007; Simon et al., 2016).

Although ozone trends have shown a decrease in the peak values due to focused efforts aimed at reducing the impact of precursors (U.S. Environmental Protection Agency (EPA), 2020a; Ahmadi & John, 2015; Simon et al., 2016), the effects of ozone at low concentrations continues to be of concern (Bell, Peng & Dominici, 2006). Further, research has shown that modeling efforts are prone to errors and are more likely to result in an underestimation of true exposure (U.S. Environmental Protection Agency (EPA), 2020b). Understanding ozone exposure is important as there is evidence to suggest a causal relationship with respiratory effects in the short term and a likely-to-be causal relationship with respiratory effects in the long term (U.S. Environmental Protection Agency (EPA), 2021b; Ji, Cohan & Bell, 2011). Efforts to estimate populations’ exposure to ozone include geostatistical and other modeling approaches that seek to estimate ozone concentrations at a variety of spatial scales and temporal durations (Liu & Rossini, 1996; Ahmadi & John, 2015). However, gaps in our understanding of how ozone impacts respiratory health continue to exist. Specifically, evaluations of seasonal long-term associations between ozone exposure and respiratory health are needed to better understand the health impacts of ozone exposure (U.S. Environmental Protection Agency (EPA), 2020b). This study develops a geostatistical model to estimate ozone concentrations over a 9-year period and examines associations between high and low levels of ozone exposures quarterly, with emergency room visits related to respiratory health diagnosis.

Pollution related health exacerbations can occur on the timescale of minutes to hours, but many previous studies aggregate the pollution data using annual means (Jerrett et al., 2009; Landrigan et al., 2018). The health impacts of air pollution exposure include both short-term symptoms like asthma or shortness of breath and long-term symptoms like respiratory illness and death (Brook et al., 2002; Szyszkowicz & Rowe, 2016). In addition, studies indicate air pollution as a risk factor for premature birth (Saldiva et al., 2018; Vieira, 2015). The combination of fine particulate matter (PM_2.5_) and ozone has been shown to cause brachial artery vasoconstriction in a timeframe as short as 2 h (Brook et al., 2002). Other studies indicate increases in the cause-specific hospital admissions due to ozone pollution include respiratory illness, asthma, and COPD (Bell et al., 2007). Additionally, many studies have identified financial benefits of reducing the air pollution burden (Caiazzo et al., 2013, Nassikas et al., 2020). Many previous health studies use risk functions (Hubbell, Fann & Levy, 2009; Burnett et al., 2014; Simon et al., 2016) while others use concentration response curves (Di et al., 2017; Berman et al., 2019; Paul et al., 2020; Wang et al., 2020). A targeted reduction strategy for ozone helps reduce other harmful precursors and has been shown to provide a financial benefit from the reduction of potential health burdens (Caiazzo et al., 2013; Carvour et al., 2018).

Many urban counties in Texas exceed the NAAQS standards for ozone. In March 2018, the Environmental Protection Agency (U.S. Environmental Protection Agency (EPA), 2018) completed its non-attainment designation for ozone and found fifteen Texas counties in non-attainment, illustrating that harmful environmental levels of pollution persist in many Texas communities (U.S. Environmental Protection Agency (EPA), 2018). Most Texas ozone non-attainment areas are urban or semi-urban. At the time of the 2010 census, over 84.7% of the Texas population lived in urban or semi-urban areas, and urban environments are growing both in size and population across Texas (Texas Demographics Center (TDC), 2020). In addition to the typical urban air pollution sources, DFW also contains many active oil and gas wells (RCT, 2021). Adverse impacts of shale activities include emissions of alkanes, especially the VOC species ethane, *n*-butane and propane, which can degrade air quality (Sather & Cavender, 2016). Another study found shale gas regions include complex interactions producing secondary pollution from fugitive natural gas that is not well understood including non-methane hydrocarbons (NMHC) also classified as VOC (Field et al., 2015). A regional DFW study investigated the relationship between ozone pollution and shale gas activities and showed that ozone pollution exposure was 8% higher in the shale gas regions (Ahmadi & John, 2015). Hays & Shonkoff (2016) reviewed over 680 papers of non-conventional natural gas development from 2009 to 2015 and reported that over 80% of these revealed results of larger pollution emissions in shale gas regions than in non-shale gas regions. This study provides a focused regional effort to better understand and quantify the variability in exposure and incidence patterns unique to DFW.

## Materials and Methods

### Study area

**Figure 1:**
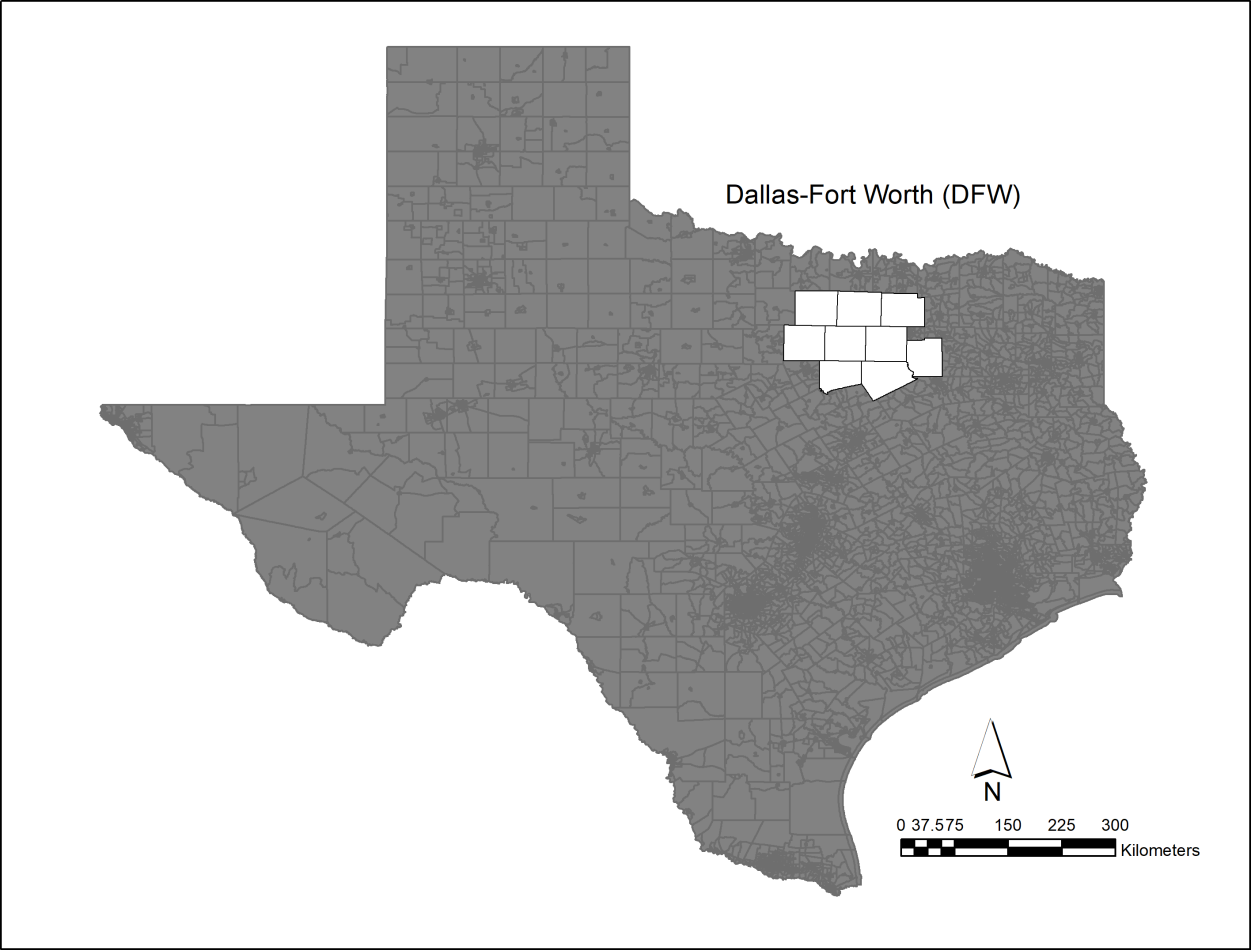
Dallas—Fort Worth study area and ozone non-attainment region (U.S. Environmental Protection Agency (EPA), 2018).

**Figure 2:**
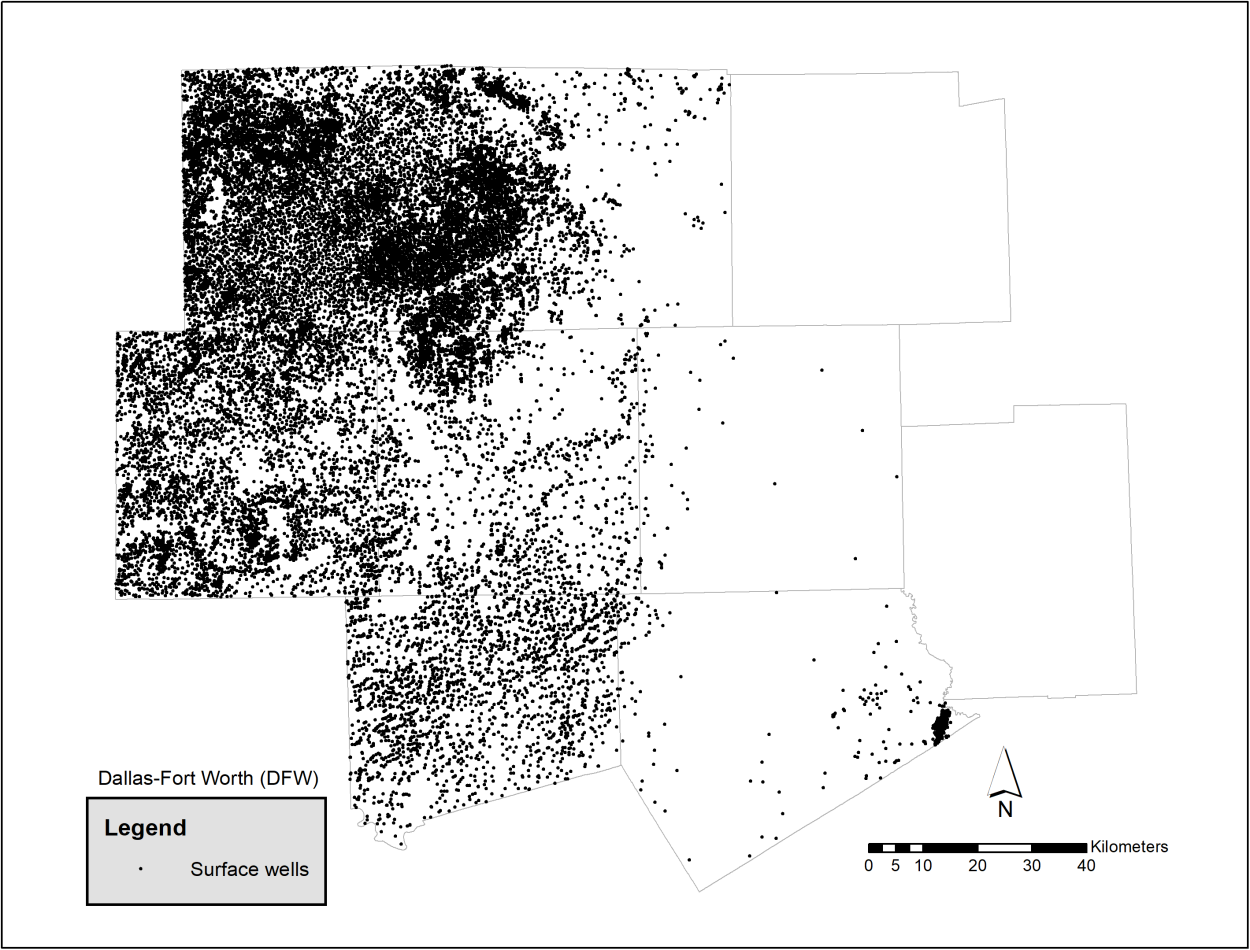
Oil and gas surface wells by county in DFW (RCT, 2021).

**Figure 3:**
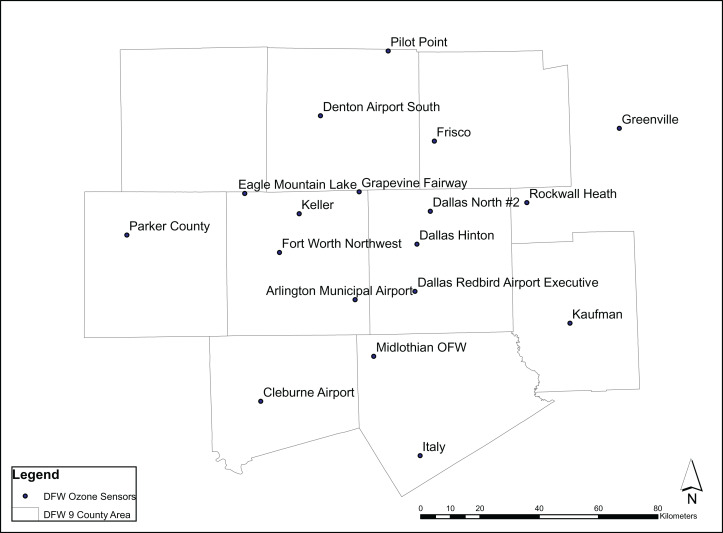
Air pollution sensor locations throughout DFW (Texas Center for Environmental Quality (TCEQ), 2020).

The study area is the Dallas-Ft Worth region, Fig. 1, and has a population of 6.2 million in 2018 (RCT, 2021) which is distributed over 7,977 mi^2^ (20,660 km^2^) and contains over 5,000 active Barnet Shale gas wells (Fig. 2). The growing population and associated energy needs in DFW, one of the fastest growing urban areas in the United States (Census, 2019), has led to pollution levels that routinely exceed acceptable health limits (U.S. Environmental Protection Agency (EPA), 2018). Sather & Cavender (2016) found in the DFW area over the last 30 years 8-h design values have seen a decline of 18 parts per billion (ppb). In addition, they found a large portion of the reduction attributed to NOx and VOC emission controls prior to 2010. Moreover, they noted that since 2010, additional NOx reductions have been observed due to the shutdown of cement kilns. In Texas, meteorological conditions for the development of ozone for the years 2011, 2012, 2015 and 2016 showed favorable conditions while 2006, 2007 were less favorable to ozone. Notably, in 2011 meteorological conditions were particularly influential due to smoke and other environmental pollutants resulting from Texas wildfires (Knowlton and Altman, 2013). In 2018, the production of natural gas is estimated to be as much as 3.176 billion ft^3^ or 89.9 million m^3^ (RCT, 2021). Figure 3 shows the ozone air pollution sensors and their locations.

### Data: sources and preprocessing

**Figure 4:**
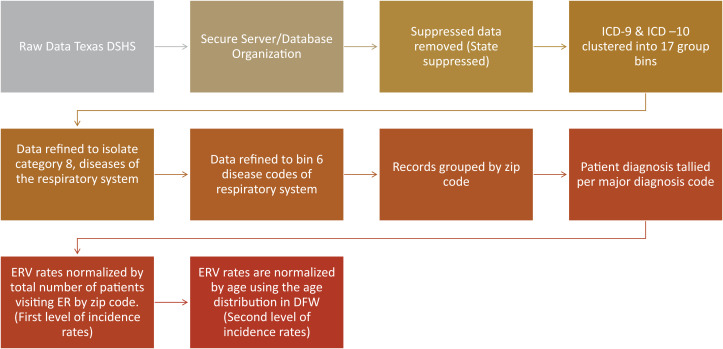
Processing flowchart for the raw data from the Texas Department of State and Health Services.

This study uses ozone and health outcome data from the EPA Air Quality Data Mart and Texas Department of State and Health Services (Texas DSHS) for the years 2007–2016. Health data from DSHS was obtained from the statewide emergency room visit (ERV) public use data file. This study required an Institutional Review Board (IRB) approval, which was obtained through the University of North Texas Cayuse IRB system (approval IRB 18-477) (Department of State Health Services of Texas (DSHS), 2018). Pre-processing of the health data was necessary and is presented in Fig. 4. The DFW study region ERV data was processed and sorted at the zip code administrative level and organized in a database. Zip code level data is suppressed from the state in multiple ways and was removed from the dataset. State suppressed data includes fewer than 30 discharges by ED, alcohol and drug use, HIV diagnosis, hospital has fewer than 50 discharges or if a hospital has fewer than five discharges of a particular gender, including unknown. Thresholds for data suppression reflect numbers per quarter.

ICD-9 and ICD-10 main diagnosis codes were first clustered into 17 major groups representing all ER diagnoses (Table 1). Then, the data was refined to focus on Category 8, diseases of the respiratory system (Table 2). This category contains seven ICD-9 and ICD-10 codes: Asthma, Bronchiectasis, Bronchitis, COPD, Emphysema, External Agents and Other. The records were grouped by zip code and the total number of patient diagnosis were tallied per major diagnosis code.

**Table 1:** Emergency room visits diagnosis codes binned by major group.

Categories	Description
0	other
1	infectious and parasitic diseases
2	neoplasms
3	endocrine, nutritional and metabolic diseases
4	diseases of the blood and blood-forming organs
5	mental disorders
6	diseases of the nervous system and sense organs
7	diseases of the circulatory system
8	diseases of the respiratory system
9	diseases of the digestive system
10	diseases of the genitourinary system
11	complications of pregnancy, childbirth, and the puerperium
12	diseases of the skin and subcutaneous tissue
13	diseases of the musculoskeletal system and connective tissue
14	congenital anomalies
15	certain conditions originating in the perinatal period
16	symptoms, signs and ill-defined conditions
17	injury and poisoning

**Table 2:** Emergency room visits diagnosis codes binned by lower respiratory disease type. Some health data zip codes were suppressed (white areas) while others were added due to access to available sensor data for a more complete ozone surface.

Lower respiratory disease categories	ICD 9 and ICD 10 codes
Asthma	493, J45
COPD (other)	496, J44
Bronchitis	466, 490, 491, J20, J21, J40, J41, J42
Emphysema	492, 506, J43
Bronchiectasis	494, J47
External Agents	495, 500, 504, 505, 506, 507, 508, J60, J61, J62, J63, J64, J65, J66, J67, J68, J69, J70
Respiratory (other)	518, 519, J22, J96, J98, J99

A limitation of the health data is that diagnosis codes were tallied instead of patients. Respiratory ERV incidence rates may be overestimated in cases where patients exhibited multiple diagnosis and were counted more than once. However, due to inconsistent practices in how I believe its ICD 9/10 codes are reported, all data points including those patients with multiple ERV codes were retained in the analysis. Further, the proportion of records with multiple diagnosis codes was small.

Our analysis was divided into three major components—the first component models ozone exposure values and ERV for respiratory outcomes across the DFW metroplex, the second component identifies spatial patterns of ozone exposures and ERV for respiratory health, and the third component uses concentration response curves to examine associations between ozone exposure and respiratory ERV.

### Component 1a: mapping the spatial patterns of respiratory ERV incidence rates

Geographic Information System software was used to create maps representing the spatial patterns of ERV incidence rates for respiratory health. ERV incidence rates for all zip codes in the study area were computed as a ratio of the total number of patients per age-group (0–18, 19–64, 65 and older) visiting the ER for respiratory health issues to the total patient visits to the ER. We do not normalize by total zip code population because we assume that ERV rates by population are skewed due to access to facilities and socioeconomic status (SES). We use the age distribution characteristics of the entire region because we assume age estimates vary within zip code over time and with large age stratification groups used in this study, the differences are negligible.

### Component 1b: mapping the spatial patterns of ozone exposures

The Air Quality (AQ) database was constructed using ozone data from the Environmental Protection Agency (EPA) Air Quality System (AQS) Data Mart for the study. For each quarter, we calculated the average daily 8-h maximum concentration for ozone from 18 monitors in the study region. This methodology was selected due to the diurnal nature of ozone exposure and to match the temporal nature of the administrative data from the Texas DSHS. In addition, the 8-h maximum exposure estimate used in this study coincides with the measurement statistic used to calculate the EPA 8-h maximum for non-compliance. The point estimates from the 18 monitors were converted to a continuous surface of ozone measurements using ordinary Kriging implemented in ArcGIS Pro. Multiple surfaces were created for each quarter during the period 2007–2016. Kriging was selected due to its wide use in estimating the ozone concentration between ambient monitoring stations (Diem, 2003; Gorai, Tchounwou & Tuluri, 2016) and its low level of measurement errors compared to inverse distance weighting and data averaging (Joseph et al., 2013). We evaluate the appropriateness of the model by constructing the semi-variogram. We selected the ordinary Kriging method and spherical semi-variogram model as it generalized well to all quarters and appeared to not over fit the surface. The mean and variance of the data were calculated and assumed to be constant over space and time. The data were assumed to be isotropic and stationary.

### Component 2: geostatistical analysis and visualization

Anselin Local Moran’s I (Evans, Love & Thurston, 2015; Al-Ahmadi, Alahmadi & Al-Zahrani, 2019; Vilinová, 2020) was used to determine spatial clusters of respiratory ERV incidence and ozone exposures for each quarter during the period 2009–2016. Cluster and outlier classification analysis aids in the discovery of spatial patterns of distinct and similar observations (Evans, Love & Thurston, 2015). The Local Moran’s I statistic evaluates the attribute value (ozone and respiratory ER visits) for each spatial unit on the map (zip code) with the values of that units’ neighbors. The level of influence (spatial weight) assigned to neighbors are a decreasing function of distance, i.e., closest neighbors are weighted more than those further away (Inverse Distance Weighted conceptualization) (Anselin, 1995). Spatial units are categorized into one of four categories: High-High (HH), High-Low (HL), Low-High (LH), and Low-Low (LL). These categories specify the relationship between every zip code on the map and its neighbors. HL and LH categories indicate spatial outliers where the observed spatial unit has a value that is different from its neighbors. HH and LL categories indicate spatial clusters where the observed spatial unit has a value like its neighbors. The output from ArcMap provides p-values for each spatial unit, thus allowing us to distinguish outliers and clusters that are statistically significant (95% confidence level).

### Component 3: concentration response curves

We created scatter plots between respiratory ERV rates vs ozone concentrations for each quarter across the study period to identify possible associations between different levels of exposure to ozone and respiratory ERV rates. For each scatter plot, a line of best fit was computed.

## Results

### Component 1: mapping the spatial patterns

The maps represent general trends in respiratory ERV incidence rates and ozone exposure for each quarter during the years 2007–08; 2011–12; and 2015–16. For each map, respiratory ERV incidence rates and ozone exposures are classified into four equal classes, where each class represents 25% of the estimated exposure value. The darkest color on the map represents those areas with the highest recorded ozone exposure value for that quarter and year. Note, however, that while the colors can be compared to assess general trends showing areas of high and low values, the numerical values (i.e., the estimated respiratory ERV rates/ozone exposure) associated with each color changes across the maps. A summary table is provided on the map panel showing the maximum and minimum values for each quartile and year. These values may be used as a guide to examine the range used by each class interval across the 24 respiratory ERV incidence and 24 ozone exposure maps.

**Figure 5:**
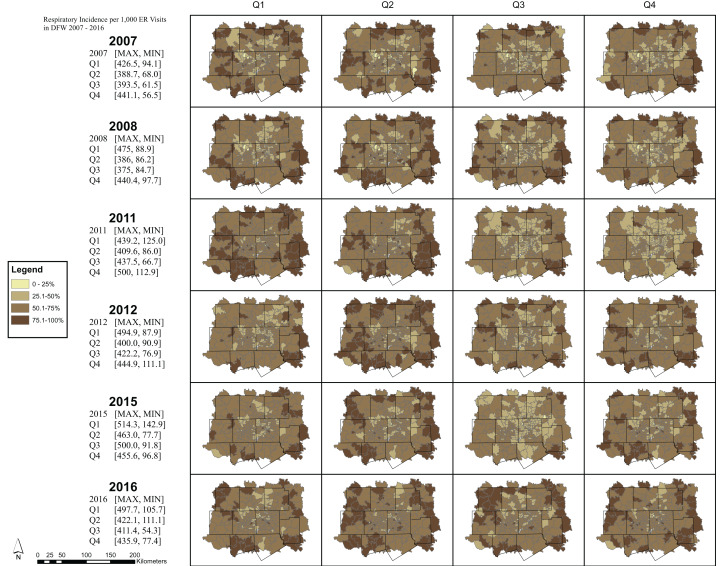
Respiratory incidence per 1,000 ER visits in DFW 2007—2016 rates by quarter. Note, however, that while the colors can be compared to assess general trends showing areas of high and low values, the numerical values (i.e., the estimated respiratory ERV rates/ozone exposure) associated with each color changes across the maps. A summary table is provided on the map panel showing the maximum and minimum values for each quartile and year.

**Figure 6:**
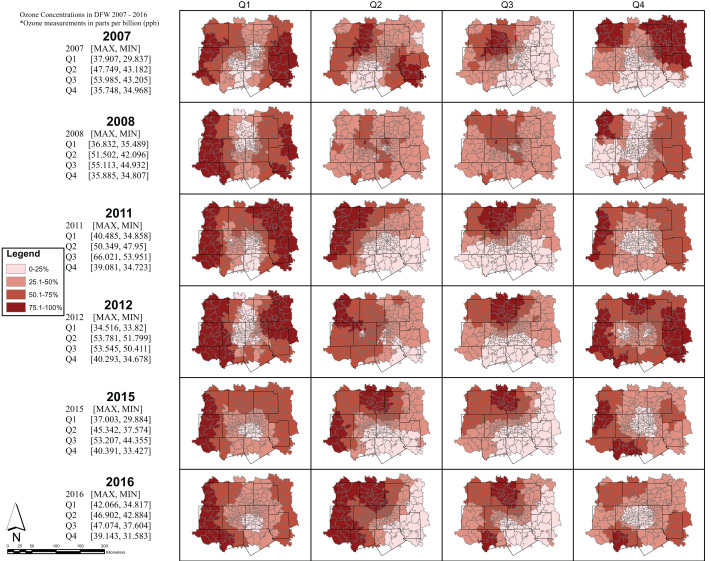
8-hour maximum ozone concentrations seasonally in DFW 2007—2016. Note, however, that while the colors can be compared to assess general trends showing areas of high and low values, the numerical values (i.e., the estimated respiratory ERV rates/ozone exposure) associated with each color change across the maps. A summary table is provided on the map panel showing the maximum and minimum values for each quarter and year.

Respiratory ERV incidence in DFW was the highest in the 1st and 4th quarters across all years (Fig. 5) while ozone exposure was highest in the 2nd and 3rd quarters (Fig. 6).

### Component 2: geostatistical analysis and visualization

**Figure 7:**
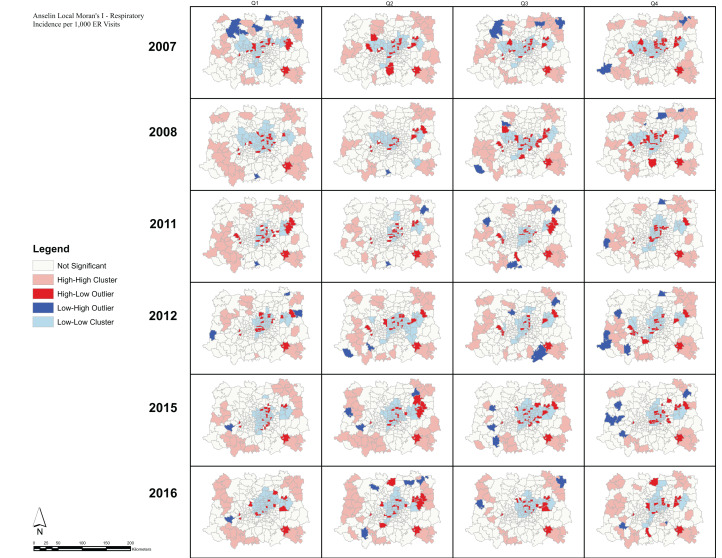
Respiratory incidence per 1,000 ER visits—Local Moran’s I. Anselin Local Moran’s I was used to determine spatial clusters of respiratory ER visit incidence for each quarter during the period 2009–2016. The Local Moran’s I statistic classifies each spatial unit on the map (zip code) into one of four categories: High-High (HH), High-Low (HL), Low-High (LH), and Low-Low (LL). These categories specify the relationship between every geographic unit on the map and its neighbors.

**Figure 8:**
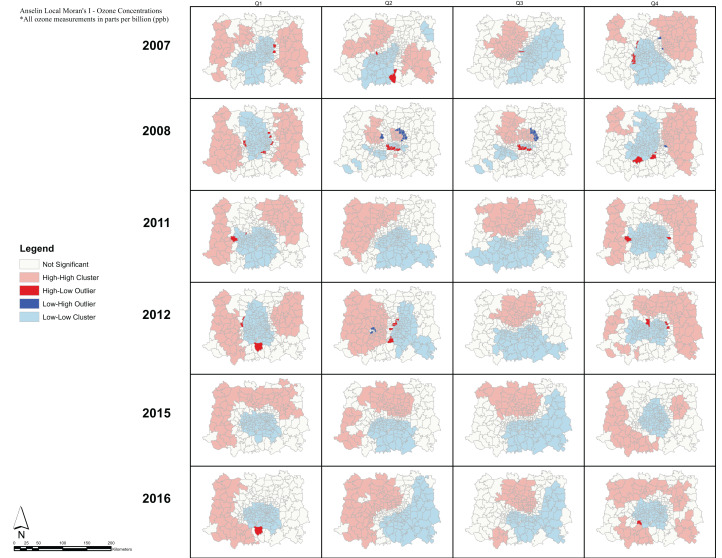
Anselin Local Moran’s I—Ozone Concentrations. Anselin Local Moran’s I was used to determine spatial clusters of ozone concentrations for each quarter during the period 2009–2016. The Local Moran’s I statistic classifies each spatial unit on the map (zip code) into one of four categories: High-High (HH), High-Low (HL), Low-High (LH), and Low-Low (LL). These categories specify the relationship between every geographic unit on the map and its neighbors, defined using the inverse distance conceptualization.

Analysis of the spatial associations of respiratory ERV incidence (Fig. 7). show HH clusters primarily outside of the urban center, while LL clusters occur almost exclusively inside the urban center. Moreover, we observe HL and LH outliers in every quarter suggesting geographic variability in the respiratory incidence observations. Note that spatial clusters of respiratory ERV incidence show similar patterns across all quarters and all years. In comparison, the spatial patterns of Moran’s I clusters for ozone exposure show consistent patterns during quarters 1 and 4 across all years, and then during quarters 2 and 3 across all years (Fig. 8). In quarters 1 and 4, HH clusters are found in the peripheral areas while LL clusters are found in the central parts of the DFW area. During quarters 2 and 3, HH clusters are found in the north and west while LL clusters are found in the south and east.

### Component 3: concentration response curves

**Figure 9:**
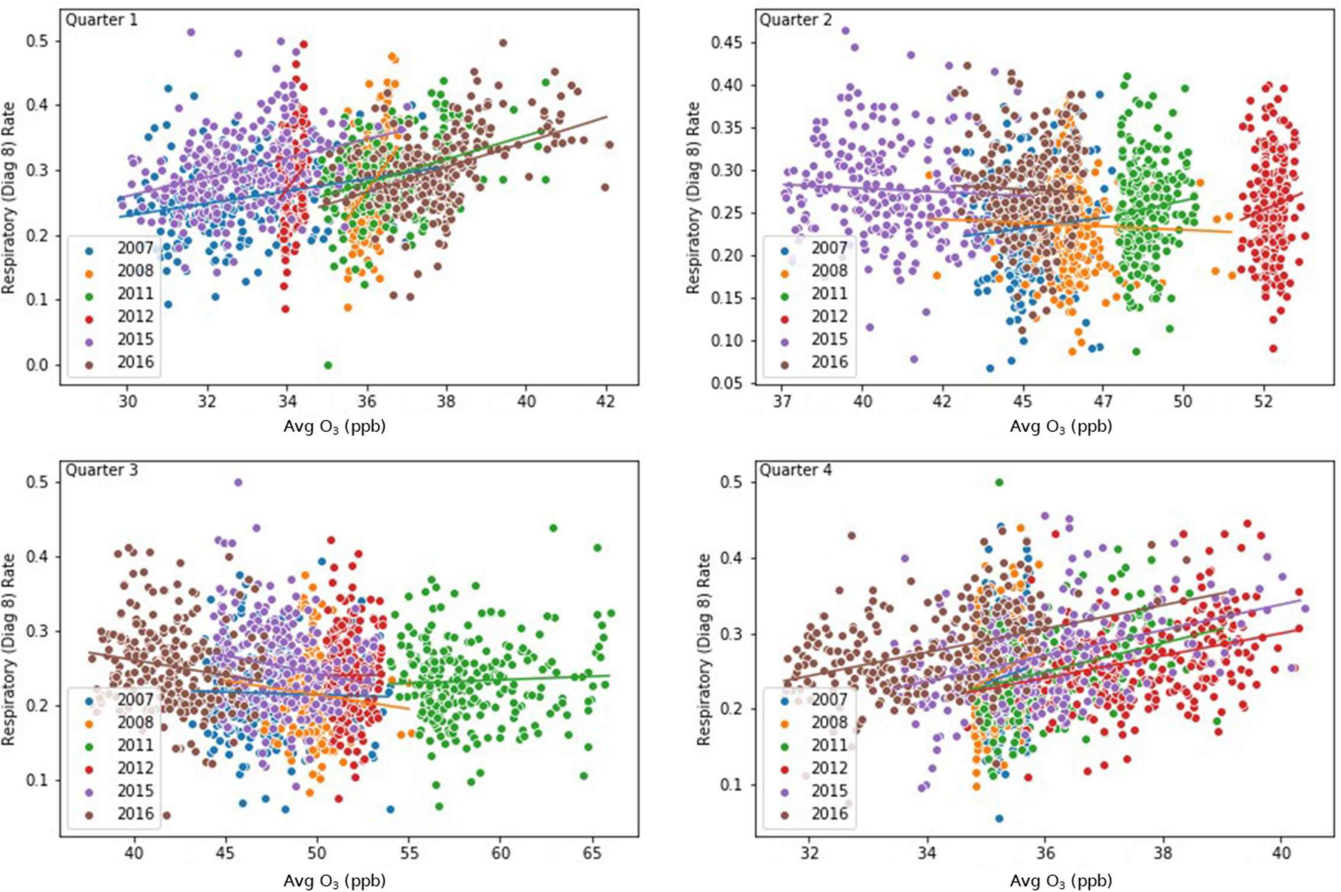
Concentration-Response Curves. Scatter plots between respiratory ER visit rates vs ozone concentrations for each quarter across the study period. A line was plotted for each year. For each scatter plot, an overall line of best fit was computed.

The scatter plots (Fig. 9) show statistically significant relationships between respiratory ERV visits and ozone exposure during all quarters with positive slopes in the 1st and 4th quarters and negative slopes in the 2nd and 3rd quarters (Table 3).

**Table 3:** Slope statistics of linear asthma/ozone response curves.

Quarter	*R*^2^	Model
Q1	0.030^**^	$\overset{˘}{Y_{Q1}}$ = 0.072 + 5.858X*_1_
Q2	0.005**	$\overset{˘}{Y_{Q2}}$ = 0.308 − 1.271X*_1_
Q3	0.005**	$\overset{˘}{Y_{Q3}}$ = 0.274 − 0.899X*_1_
Q4	0.022**	$\overset{˘}{Y_{Q4}}$ = 0.009 + 6.769X*_1_

**Notes:**

Ozone (X_1_), Respiratory ERV (Y).

* *p* < 0.01 for β_1._

** *p* < 0.01, α = 0.05.

Each quarter shows an exposure-response curve equation for respiratory incidence rates (*y*) and ozone exposure (*x*).

## Discussion

In this study, we examined the spatial clustering and variations of ozone exposure and respiratory ERV incidence rates in DFW over a nine-year period using 266 zip codes. Mapping the spatial patterns found respiratory ERV incidence was highest primarily in the 1st and 4th quarters while ozone exposure was highest in the 2nd and 3rd quarters. At the time of this publication, we can find study no study to compare or contrast these results. The ozone finding is consistent with literature indicating quarters 2 and 3 are often the largest exposures, occurring during the maximum values of incoming solar radiation and favorable meteorological conditions for ozone formation (Simon et al., 2015; Simon et al., 2016; Kim, Byun & Cohan, 2009).

Conclusions drawn from the Anselin Local Moran’s I analysis are that significant clusters have higher or lower rates than expected from a random distribution. High and low clusters reflect higher or lower than expected clustering of values, respectively. A cluster and outlier analysis and visualization for the respiratory ERV data shows the presence of HL and LH outliers throughout the DFW metroplex. On the other hand, a similar analysis of ozone exposure reveals consistent patterns of HH and LL cluster values. For ozone, the HH clusters tend to be in the northern and western parts of the DFW area during the high season (Q2 and Q3). This is expected given that the ozone transport and precursor distribution is influenced by the prevailing winds in the region being from SSE to NNW, thus causing an increased development of ozone in the observed clusters (Kim, Byun & Cohan, 2009). Meanwhile, these areas of HH and LL clusters present compelling indicators of large-scale spatial regions with high or low levels of ozone than expected from a random distribution. Our study shows that there seems to be no direct correlation in the spatial patterns of respiratory ERV incidence rates and ozone exposure. This result can be partially explained due to the lack of extensive ground monitoring sites and the use of a geostatistical model to estimate ozone exposure across the DFW area (U.S. Environmental Protection Agency (EPA), 2020b). Note that the use of such models is common in studies of air pollution modeling (Liu & Rossini, 1996; Diem, 2003; Gorai, Tchounwou & Tuluri, 2016).

Exposure-response curves for respiratory incidence rates and ozone exposure indicate significant slope coefficients with +β_1_ in Q1 and Q4 and −β_1_ in Q2 and Q3. Note that, the predictive value was low for ozone on respiratory health (*R*^2^ < 0.031) for all quarters. Despite the low *R*^2^ values, the study found that ozone was statistically significant across all quarters but highest in quarters 1 and 4. This finding is consistent with the cluster and outlier analysis in the respective quarters as well as the positive slope coefficients. This suggests that ozone can affect respiratory health at levels below the 70-ppb ozone standard (Bell, Peng & Dominici, 2006), and therefore presents evidence that lower ozone levels are influencing long-term respiratory illness. We acknowledge this study did not consider co-pollutant confounding in our single pollutant model. Although this uncertainty exists, we point out the U.S. Environmental Protection Agency (EPA) (2020b) sections 2.5 & 3.2.5.1 found co-pollutant correlations with ozone to be low, and less of a concern in the design of ozone and health effects studies. Moreover, population mobility was also excluded in the analysis which may influence interpretation of the results. Finally, the authors acknowledge this study has the potential to be capturing an accumulation of the short-term acute impacts of ozone on adverse respiratory events and may not be able to conceptually separate those outcomes that are a result of long-term chronic effects.

## Conclusions

The spatial distribution of ozone exposure has public health implications. This study examined the spatial clusters and outliers of ozone exposure and respiratory incidence rates in DFW using emergency room visits and ozone monitoring data from 2007 to 2016. Evidence presented shows no spatial relationships between zip code level respiratory ERV incidence rates and ozone exposure but found statistically significant widespread spatially influenced clusters of ozone. In addition, a relationship between ozone exposure and respiratory health during Q1 and Q4 was noted in DFW. The results of this study contribute to the understanding of the relationships between respiratory health outcomes and long-term exposure to ozone. Our results suggest that prioritization of targeted pollutant reduction interventions, even during times of low exposure, are important to minimize health impacts.

## Supplemental Information

10.7717/peerj.11066/supp-1 Supplemental Information 1 Average 8-hour daily ozone concentrations by quarter from 2007–2016. Each data point contains the quarterly 8-hour daily average by year and quarter for each of the eighteen sensors in the DFW study area.

10.7717/peerj.11066/supp-2 Supplemental Information 2 Statistics for ERV data.

10.7717/peerj.11066/supp-3 Supplemental Information 3 Respiratory incidence rates and quarterly differences. The data contained is a summary of the respiratory incidence rates as found in Figure 7.

10.7717/peerj.11066/supp-4 Supplemental Information 4 Ozone exposures and differences quarterly. The data is a summary of the ozone exposures as visualized in Figure 8.

10.7717/peerj.11066/supp-5 Supplemental Information 5 Processed ERV dataset by diagnostic code by quarter by zipcode. This is the processed ERV datasets approved for release by the contractual owner of the data, the Texas DSHS. This data set has been adequately suppressed per the DSHS and processed through the second level of incidence as shown in Figure 4.

## Additional Information and Declarations

### Data Availability

The following information was supplied regarding data availability: Raw data are available in the Supplemental Files. Statewide emergency room visit (ERV) public use data file can obtained from the Texas Department of State and Health Services (Texas DSHS) for the years 2007–2016 after first obtaining Institutional Review Board (IRB) approval.
