# Supplementary material for: Evaluating spatial patterns of seasonal ozone exposure and incidence of respiratory emergency room visits in Dallas-Fort Worth
Source: PeerJ. 2021 Apr 13;9:e11066. doi: 10.7717/peerj.11066 (PMC8051349; doi:10.7717/peerj.11066)
Supplement: Supplemental Information 3 — The data contained is a summary of the respiratory incidence rates as found in Figure 7. [file peerj-09-11066-s003.pdf]

Respiratory Incidence (per 1,000)					
		1Q	2Q	3Q	4Q
2007	H:	426.5	388.7	393.5	441.4
	L:	94.1	68.0	61.5	56.5
2008	H:	475.0	386.0	375.0	440.4
	L:	88.9	86.2	84.7	97.9
2011	H:	439.2	409.6	437.5	500.0
	L:	125.0	86.0	66.7	112.9
2012	H:	494.9	400.0	422.2	444.9
	L:	87.9	90.9	76.9	111.1
2015	H:	514.3	463.0	500.0	455.6
	L:	142.9	77.7	91.8	96.8
2016	H:	497.7	422.1	411.4	435.9
	L:	105.7	111.1	54.3	77.4
Respiratory Incidence Difference (per 1,000)					
		1Q	2Q	3Q	4Q
2007	D:	332.4	320.7	332.0	384.9
2008	D:	386.1	299.8	290.3	342.5
2011	D:	314.2	323.6	370.8	387.1
2012	D:	407.1	309.1	345.3	333.8
2015	D:	371.4	385.3	408.2	358.8
2016	D:	391.9	311.0	357.1	358.5
Average		367.2	324.9	350.6	360.9
